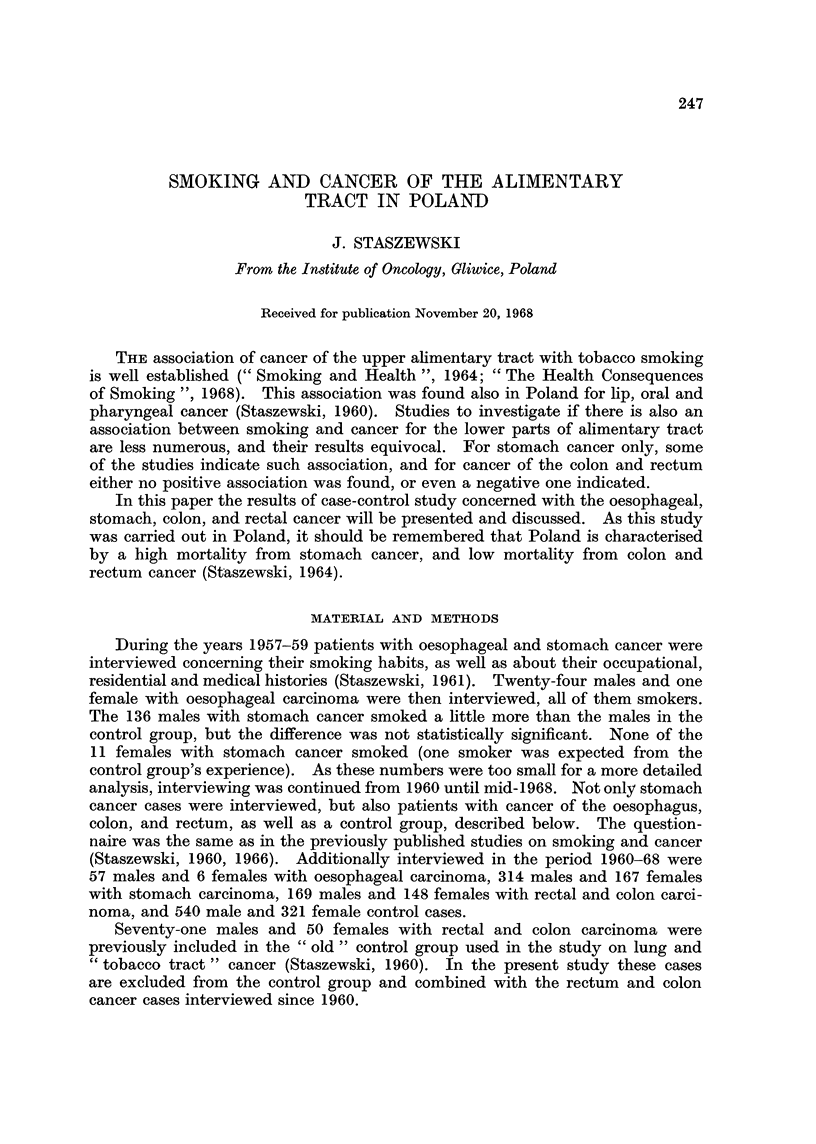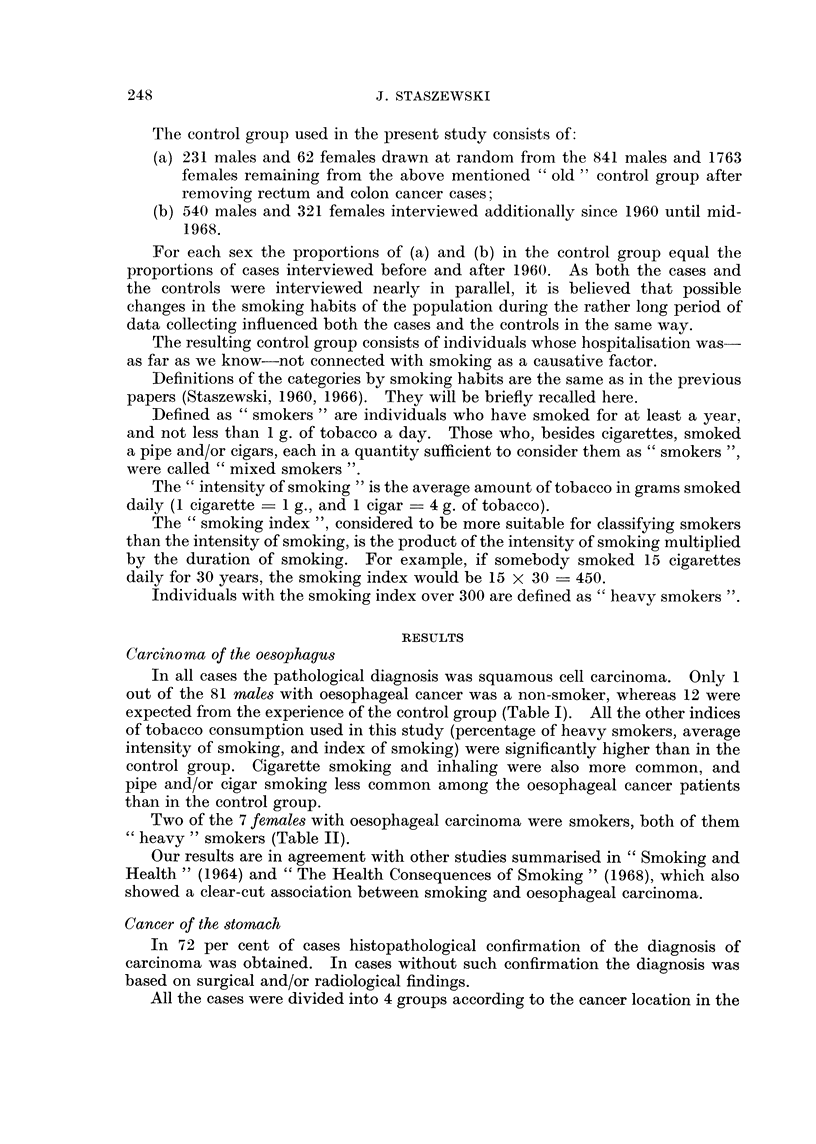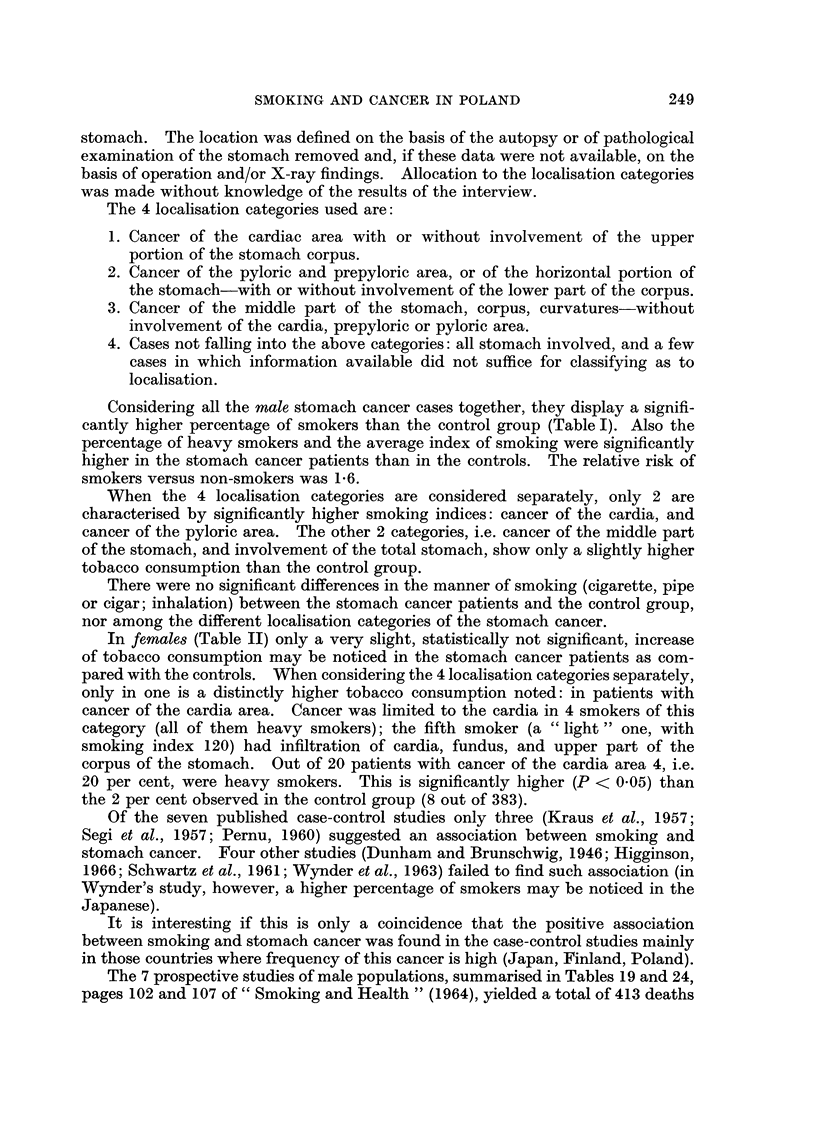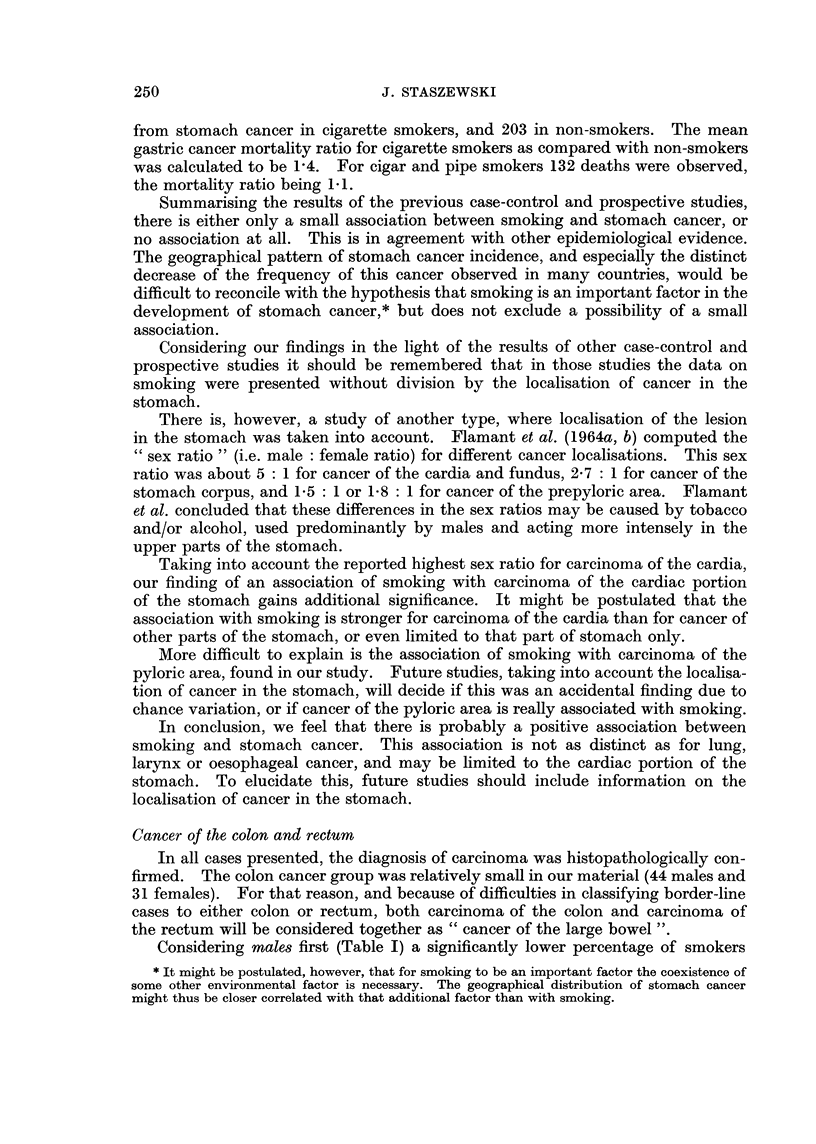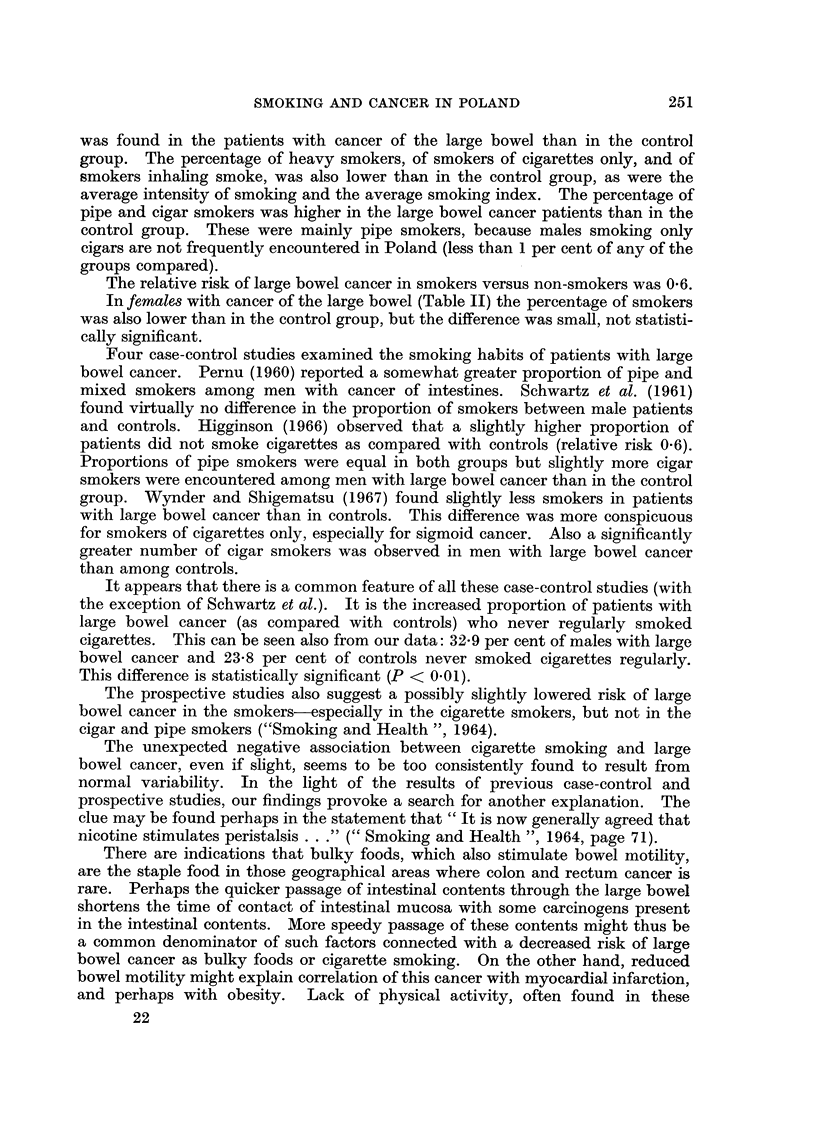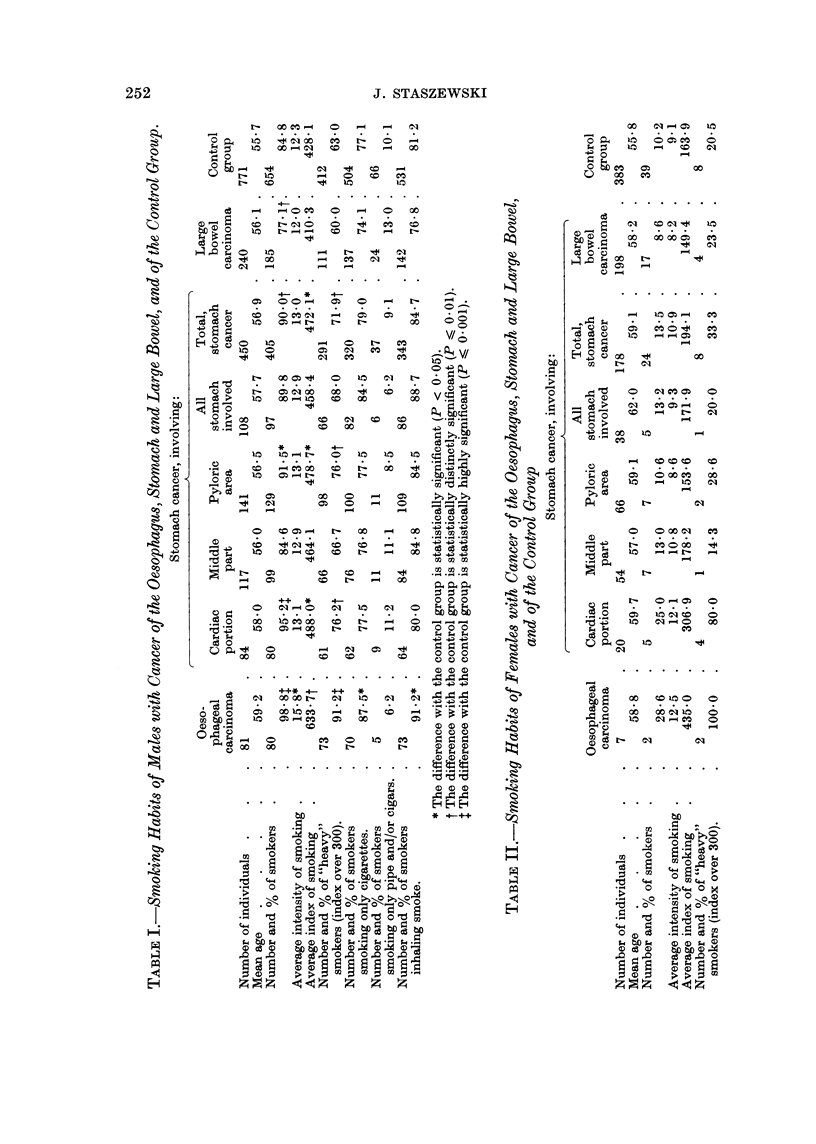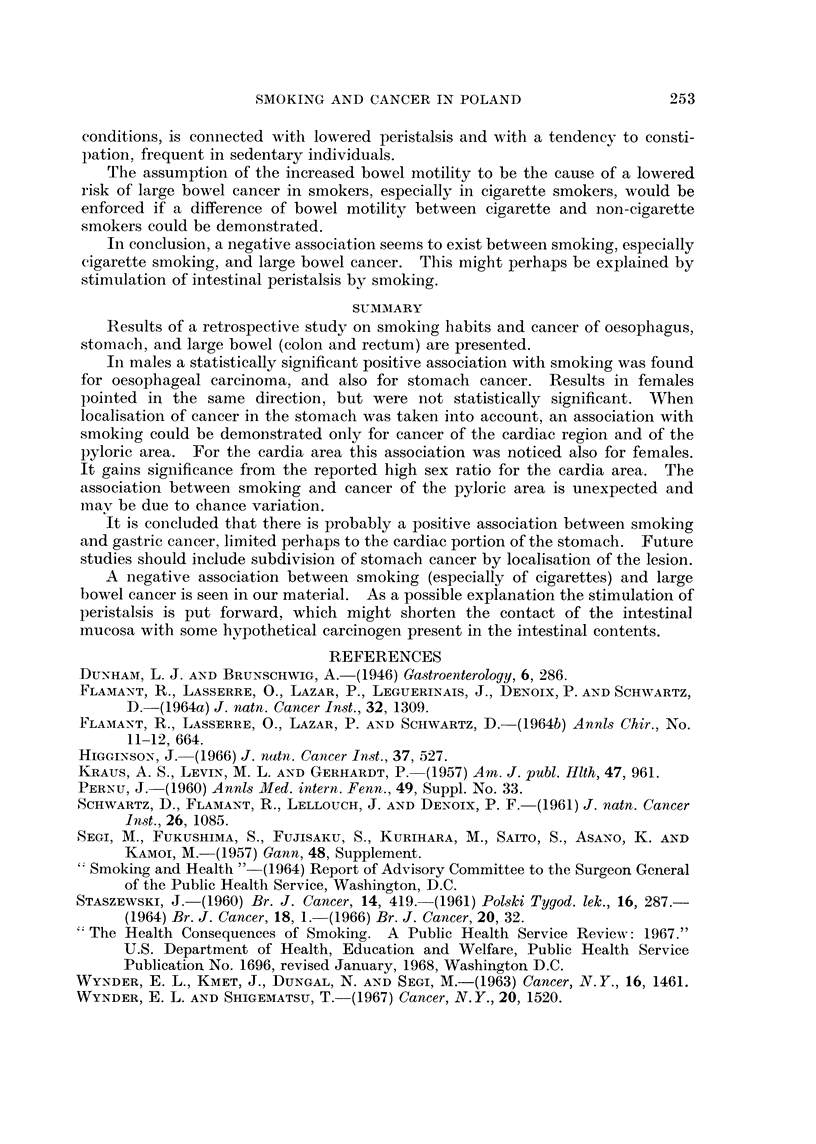# Smoking and cancer of the alimentary tract in Poland.

**DOI:** 10.1038/bjc.1969.34

**Published:** 1969-06

**Authors:** J. Staszewski


### SMOKING AND CANCER OF THE ALIMENTARY TRACT IN POLAND

J. STASZEWSKI

*From the Institute of Oncology, Gliwice, Poland*

Received for publication November 20, 1968

THE association of cancer of the upper alimentary tract with tobacco smoking is well established (" Smoking and Health ", 1964; " The Health Consequences of Smoking ", 1968). This association was found also in Poland for lip, oral and pharyngeal cancer (Staszewski, 1960). Studies to investigate if there is also an association between smoking and cancer for the lower parts of alimentary tract are less numerous, and their results equivocal. For stomach cancer only, some of the studies indicate such association, and for cancer of the colon and rectum either no positive association was found, or even a negative one indicated.

In this paper the results of case-control study concerned with the oesophageal, stomach, colon, and rectal cancer will be presented and discussed. As this study was carried out in Poland, it should be remembered that Poland is characterised by a high mortality from stomach cancer, and low mortality from colon and rectum cancer (Staszewski, 1964).

### MATERIAL AND METHODS

During the years 1957–59 patients with oesophageal and stomach cancer were interviewed concerning their smoking habits, as well as about their occupational, residential and medical histories (Staszewski, 1961). Twenty-four males and one female with oesophageal carcinoma were then interviewed, all of them smokers. The 136 males with stomach cancer smoked a little more than the males in the control group, but the difference was not statistically significant. None of the 11 females with stomach cancer smoked (one smoker was expected from the control group's experience). As these numbers were too small for a more detailed analysis, interviewing was continued from 1960 until mid-1968. Not only stomach cancer cases were interviewed, but also patients with cancer of the oesophagus, colon, and rectum, as well as a control group, described below. The questionnaire was the same as in the previously published studies on smoking and cancer (Staszewski, 1960, 1966). Additionally interviewed in the period 1960–68 were 57 males and 6 females with oesophageal carcinoma, 314 males and 167 females with stomach carcinoma, 169 males and 148 females with rectal and colon carcinoma, and 540 male and 321 female control cases.

Seventy-one males and 50 females with rectal and colon carcinoma were previously included in the " old " control group used in the study on lung and " tobacco tract " cancer (Staszewski, 1960). In the present study these cases are excluded from the control group and combined with the rectum and colon cancer cases interviewed since 1960.

The control group used in the present study consists of:

(a) 231 males and 62 females drawn at random from the 841 males and 1763 females remaining from the above mentioned " old " control group after removing rectum and colon cancer cases;

(b) 540 males and 321 females interviewed additionally since 1960 until mid-1968.

For each sex the proportions of (a) and (b) in the control group equal the proportions of cases interviewed before and after 1960. As both the cases and the controls were interviewed nearly in parallel, it is believed that possible changes in the smoking habits of the population during the rather long period of data collecting influenced both the cases and the controls in the same way.

The resulting control group consists of individuals whose hospitalisation was—as far as we know—not connected with smoking as a causative factor.

Definitions of the categories by smoking habits are the same as in the previous papers (Staszewski, 1960, 1966). They will be briefly recalled here.

Defined as " smokers " are individuals who have smoked for at least a year, and not less than 1 g. of tobacco a day. Those who, besides cigarettes, smoked a pipe and/or cigars, each in a quantity sufficient to consider them as " smokers ", were called " mixed smokers ".

The " intensity of smoking " is the average amount of tobacco in grams smoked daily (1 cigarette = 1 g., and 1 cigar = 4 g. of tobacco).

The " smoking index ", considered to be more suitable for classifying smokers than the intensity of smoking, is the product of the intensity of smoking multiplied by the duration of smoking. For example, if somebody smoked 15 cigarettes daily for 30 years, the smoking index would be $15 \times 30 = 450$.

Individuals with the smoking index over 300 are defined as " heavy smokers ".

### RESULTS

### *Carcinoma of the oesophagus*

In all cases the pathological diagnosis was squamous cell carcinoma. Only 1 out of the 81 *males* with oesophageal cancer was a non-smoker, whereas 12 were expected from the experience of the control group (Table I). All the other indices of tobacco consumption used in this study (percentage of heavy smokers, average intensity of smoking, and index of smoking) were significantly higher than in the control group. Cigarette smoking and inhaling were also more common, and pipe and/or cigar smoking less common among the oesophageal cancer patients than in the control group.

Two of the 7 *females* with oesophageal carcinoma were smokers, both of them " heavy " smokers (Table II).

Our results are in agreement with other studies summarised in " Smoking and Health " (1964) and " The Health Consequences of Smoking " (1968), which also showed a clear-cut association between smoking and oesophageal carcinoma.

### *Cancer of the stomach*

In 72 per cent of cases histopathological confirmation of the diagnosis of carcinoma was obtained. In cases without such confirmation the diagnosis was based on surgical and/or radiological findings.

All the cases were divided into 4 groups according to the cancer location in the stomach. The location was defined on the basis of the autopsy or of pathological examination of the stomach removed and, if these data were not available, on the basis of operation and/or X-ray findings. Allocation to the localisation categories was made without knowledge of the results of the interview.

The 4 localisation categories used are:

1. Cancer of the cardiac area with or without involvement of the upper portion of the stomach corpus.
2. Cancer of the pyloric and prepyloric area, or of the horizontal portion of the stomach—with or without involvement of the lower part of the corpus.
3. Cancer of the middle part of the stomach, corpus, curvatures—without involvement of the cardia, prepyloric or pyloric area.
4. Cases not falling into the above categories: all stomach involved, and a few cases in which information available did not suffice for classifying as to localisation.

Considering all the *male* stomach cancer cases together, they display a significantly higher percentage of smokers than the control group (Table I). Also the percentage of heavy smokers and the average index of smoking were significantly higher in the stomach cancer patients than in the controls. The relative risk of smokers versus non-smokers was 1·6.

When the 4 localisation categories are considered separately, only 2 are characterised by significantly higher smoking indices: cancer of the cardia, and cancer of the pyloric area. The other 2 categories, i.e. cancer of the middle part of the stomach, and involvement of the total stomach, show only a slightly higher tobacco consumption than the control group.

There were no significant differences in the manner of smoking (cigarette, pipe or cigar; inhalation) between the stomach cancer patients and the control group, nor among the different localisation categories of the stomach cancer.

In *females* (Table II) only a very slight, statistically not significant, increase of tobacco consumption may be noticed in the stomach cancer patients as compared with the controls. When considering the 4 localisation categories separately, only in one is a distinctly higher tobacco consumption noted: in patients with cancer of the cardia area. Cancer was limited to the cardia in 4 smokers of this category (all of them heavy smokers); the fifth smoker (a " light " one, with smoking index 120) had infiltration of cardia, fundus, and upper part of the corpus of the stomach. Out of 20 patients with cancer of the cardia area 4, i.e. 20 per cent, were heavy smokers. This is significantly higher ($P < 0{\cdot}05$) than the 2 per cent observed in the control group (8 out of 383).

Of the seven published case-control studies only three (Kraus *et al.*, 1957; Segi *et al.*, 1957; Pernu, 1960) suggested an association between smoking and stomach cancer. Four other studies (Dunham and Brunschwig, 1946; Higginson, 1966; Schwartz *et al.*, 1961; Wynder *et al.*, 1963) failed to find such association (in Wynder's study, however, a higher percentage of smokers may be noticed in the Japanese).

It is interesting if this is only a coincidence that the positive association between smoking and stomach cancer was found in the case-control studies mainly in those countries where frequency of this cancer is high (Japan, Finland, Poland).

The 7 prospective studies of male populations, summarised in Tables 19 and 24, pages 102 and 107 of " Smoking and Health " (1964), yielded a total of 413 deaths from stomach cancer in cigarette smokers, and 203 in non-smokers. The mean gastric cancer mortality ratio for cigarette smokers as compared with non-smokers was calculated to be 1·4. For cigar and pipe smokers 132 deaths were observed, the mortality ratio being 1·1.

Summarising the results of the previous case-control and prospective studies, there is either only a small association between smoking and stomach cancer, or no association at all. This is in agreement with other epidemiological evidence. The geographical pattern of stomach cancer incidence, and especially the distinct decrease of the frequency of this cancer observed in many countries, would be difficult to reconcile with the hypothesis that smoking is an important factor in the development of stomach cancer,* but does not exclude a possibility of a small association.

Considering our findings in the light of the results of other case-control and prospective studies it should be remembered that in those studies the data on smoking were presented without division by the localisation of cancer in the stomach.

There is, however, a study of another type, where localisation of the lesion in the stomach was taken into account. Flamant *et al.* (1964*a*, *b*) computed the " sex ratio " (i.e. male : female ratio) for different cancer localisations. This sex ratio was about 5 : 1 for cancer of the cardia and fundus, 2·7 : 1 for cancer of the stomach corpus, and 1·5 : 1 or 1·8 : 1 for cancer of the prepyloric area. Flamant *et al.* concluded that these differences in the sex ratios may be caused by tobacco and/or alcohol, used predominantly by males and acting more intensely in the upper parts of the stomach.

Taking into account the reported highest sex ratio for carcinoma of the cardia, our finding of an association of smoking with carcinoma of the cardiac portion of the stomach gains additional significance. It might be postulated that the association with smoking is stronger for carcinoma of the cardia than for cancer of other parts of the stomach, or even limited to that part of stomach only.

More difficult to explain is the association of smoking with carcinoma of the pyloric area, found in our study. Future studies, taking into account the localisation of cancer in the stomach, will decide if this was an accidental finding due to chance variation, or if cancer of the pyloric area is really associated with smoking.

In conclusion, we feel that there is probably a positive association between smoking and stomach cancer. This association is not as distinct as for lung, larynx or oesophageal cancer, and may be limited to the cardiac portion of the stomach. To elucidate this, future studies should include information on the localisation of cancer in the stomach.

*Cancer of the colon and rectum*

In all cases presented, the diagnosis of carcinoma was histopathologically confirmed. The colon cancer group was relatively small in our material (44 males and 31 females). For that reason, and because of difficulties in classifying border-line cases to either colon or rectum, both carcinoma of the colon and carcinoma of the rectum will be considered together as " cancer of the large bowel ".

Considering *males* first (Table I) a significantly lower percentage of smokers

* It might be postulated, however, that for smoking to be an important factor the coexistence of some other environmental factor is necessary. The geographical distribution of stomach cancer might thus be closer correlated with that additional factor than with smoking.

was found in the patients with cancer of the large bowel than in the control group. The percentage of heavy smokers, of smokers of cigarettes only, and of smokers inhaling smoke, was also lower than in the control group, as were the average intensity of smoking and the average smoking index. The percentage of pipe and cigar smokers was higher in the large bowel cancer patients than in the control group. These were mainly pipe smokers, because males smoking only cigars are not frequently encountered in Poland (less than 1 per cent of any of the groups compared).

The relative risk of large bowel cancer in smokers versus non-smokers was 0·6.

In *females* with cancer of the large bowel (Table II) the percentage of smokers was also lower than in the control group, but the difference was small, not statistically significant.

Four case-control studies examined the smoking habits of patients with large bowel cancer. Pernu (1960) reported a somewhat greater proportion of pipe and mixed smokers among men with cancer of intestines. Schwartz *et al.* (1961) found virtually no difference in the proportion of smokers between male patients and controls. Higginson (1966) observed that a slightly higher proportion of patients did not smoke cigarettes as compared with controls (relative risk 0·6). Proportions of pipe smokers were equal in both groups but slightly more cigar smokers were encountered among men with large bowel cancer than in the control group. Wynder and Shigematsu (1967) found slightly less smokers in patients with large bowel cancer than in controls. This difference was more conspicuous for smokers of cigarettes only, especially for sigmoid cancer. Also a significantly greater number of cigar smokers was observed in men with large bowel cancer than among controls.

It appears that there is a common feature of all these case-control studies (with the exception of Schwartz *et al.*). It is the increased proportion of patients with large bowel cancer (as compared with controls) who never regularly smoked cigarettes. This can be seen also from our data: 32·9 per cent of males with large bowel cancer and 23·8 per cent of controls never smoked cigarettes regularly. This difference is statistically significant ($P < 0{\cdot}01$).

The prospective studies also suggest a possibly slightly lowered risk of large bowel cancer in the smokers—especially in the cigarette smokers, but not in the cigar and pipe smokers ("Smoking and Health ", 1964).

The unexpected negative association between cigarette smoking and large bowel cancer, even if slight, seems to be too consistently found to result from normal variability. In the light of the results of previous case-control and prospective studies, our findings provoke a search for another explanation. The clue may be found perhaps in the statement that " It is now generally agreed that nicotine stimulates peristalsis . . ." (" Smoking and Health ", 1964, page 71).

There are indications that bulky foods, which also stimulate bowel motility, are the staple food in those geographical areas where colon and rectum cancer is rare. Perhaps the quicker passage of intestinal contents through the large bowel shortens the time of contact of intestinal mucosa with some carcinogens present in the intestinal contents. More speedy passage of these contents might thus be a common denominator of such factors connected with a decreased risk of large bowel cancer as bulky foods or cigarette smoking. On the other hand, reduced bowel motility might explain correlation of this cancer with myocardial infarction, and perhaps with obesity. Lack of physical activity, often found in these

TABLE I.—*Smoking Habits of Males with Cancer of the Oesophagus, Stomach and Large Bowel, and of the Control Group.*

| | Oeso-phageal carcinoma | Stomach cancer, involving: Cardiac portion | Middle part | Pyloric area | All stomach involved | Total, stomach cancer | Large bowel carcinoma | Control group |
|---|---|---|---|---|---|---|---|---|
| Number of individuals | 81 | 84 | 117 | 141 | 108 | 450 | 240 | 771 |
| Mean age | 59·2 | 58·0 | 56·0 | 56·5 | 57·7 | 56·9 | 56·1 | 55·7 |
| Number and % of smokers | 80 | 80 | 99 | 129 | 97 | 405 | 185 | 654 |
| | 98·8‡ | 95·2‡ | 84·6 | 91·5* | 89·8 | 90·0† | 77·1† | 84·8 |
| Average intensity of smoking | 15·8* | 13·1 | 12·9 | 13·1 | 12·9 | 13·0 | 12·0 | 12·3 |
| Average index of smoking | 633·7† | 488·0* | 464·1 | 478·7* | 458·4 | 472·1* | 410·3 | 428·1 |
| Number and % of "heavy" | 73 | 61 | 66 | 98 | 66 | 291 | 111 | 412 |
| smokers (index over 300). | 91·2‡ | 76·2† | 66·7 | 76·0† | 68·0 | 71·9† | 60·0 | 63·0 |
| Number and % of smokers | 70 | 62 | 76 | 100 | 82 | 320 | 137 | 504 |
| smoking only cigarettes. | 87·5* | 77·5 | 76·8 | 77·5 | 84·5 | 79·0 | 74·1 | 77·1 |
| Number and % of smokers | 5 | 9 | 11 | 11 | 6 | 37 | 24 | 66 |
| smoking only pipe and/or cigars. | 6·2 | 11·2 | 11·1 | 8·5 | 6·2 | 9·1 | 13·0 | 10·1 |
| Number and % of smokers | 73 | 64 | 84 | 109 | 86 | 343 | 142 | 531 |
| inhaling smoke. | 91·2* | 80·0 | 84·8 | 84·5 | 88·7 | 84·7 | 76·8 | 81·2 |

* The difference with the control group is statistically significant ($P < 0\cdot05$).

† The difference with the control group is statistically distinctly significant ($P \leqslant 0\cdot01$).

‡ The difference with the control group is statistically highly significant ($P \leqslant 0\cdot001$).

TABLE II.—*Smoking Habits of Females with Cancer of the Oesophagus, Stomach and Large Bowel, and of the Control Group*

| | Oesophageal carcinoma | Stomach cancer, involving: Cardiac portion | Middle part | Pyloric area | All stomach involved | Total, stomach cancer | Large bowel carcinoma | Control group |
|---|---|---|---|---|---|---|---|---|
| Number of individuals | 7 | 20 | 54 | 66 | 38 | 178 | 198 | 383 |
| Mean age | 58·8 | 59·7 | 57·0 | 59·1 | 62·0 | 59·1 | 58·2 | 55·8 |
| Number and % of smokers | 2 | 5 | 7 | 7 | 5 | 24 | 17 | 39 |
| | 28·6 | 25·0 | 13·0 | 10·6 | 13·2 | 13·5 | 8·6 | 10·2 |
| Average intensity of smoking | 12·5 | 12·1 | 10·8 | 8·6 | 9·3 | 10·9 | 8·2 | 9·1 |
| Average index of smoking | 435·0 | 306·9 | 178·2 | 153·6 | 171·9 | 194·1 | 149·4 | 163·9 |
| Number and % of "heavy" | 2 | 4 | 1 | 2 | 1 | 8 | 4 | 8 |
| smokers (index over 300). | 100·0 | 80·0 | 14·3 | 28·6 | 20·0 | 33·3 | 23·5 | 20·5 |

conditions, is connected with lowered peristalsis and with a tendency to constipation, frequent in sedentary individuals.

The assumption of the increased bowel motility to be the cause of a lowered risk of large bowel cancer in smokers, especially in cigarette smokers, would be enforced if a difference of bowel motility between cigarette and non-cigarette smokers could be demonstrated.

In conclusion, a negative association seems to exist between smoking, especially cigarette smoking, and large bowel cancer. This might perhaps be explained by stimulation of intestinal peristalsis by smoking.

SUMMARY

Results of a retrospective study on smoking habits and cancer of oesophagus, stomach, and large bowel (colon and rectum) are presented.

In males a statistically significant positive association with smoking was found for oesophageal carcinoma, and also for stomach cancer. Results in females pointed in the same direction, but were not statistically significant. When localisation of cancer in the stomach was taken into account, an association with smoking could be demonstrated only for cancer of the cardiac region and of the pyloric area. For the cardia area this association was noticed also for females. It gains significance from the reported high sex ratio for the cardia area. The association between smoking and cancer of the pyloric area is unexpected and may be due to chance variation.

It is concluded that there is probably a positive association between smoking and gastric cancer, limited perhaps to the cardiac portion of the stomach. Future studies should include subdivision of stomach cancer by localisation of the lesion.

A negative association between smoking (especially of cigarettes) and large bowel cancer is seen in our material. As a possible explanation the stimulation of peristalsis is put forward, which might shorten the contact of the intestinal mucosa with some hypothetical carcinogen present in the intestinal contents.

REFERENCES

DUNHAM, L. J. AND BRUNSCHWIG, A.—(1946) *Gastroenterology*, **6**, 286.

FLAMANT, R., LASSERRE, O., LAZAR, P., LEGUERINAIS, J., DENOIX, P. AND SCHWARTZ, D.—(1964*a*) *J. natn. Cancer Inst.*, **32**, 1309.

FLAMANT, R., LASSERRE, O., LAZAR, P. AND SCHWARTZ, D.—(1964*b*) *Annls Chir.*, No. 11–12, 664.

HIGGINSON, J.—(1966) *J. natn. Cancer Inst.*, **37**, 527.

KRAUS, A. S., LEVIN, M. L. AND GERHARDT, P.—(1957) *Am. J. publ. Hlth*, **47**, 961.

PERNU, J.—(1960) *Annls Med. intern. Fenn.*, **49**, Suppl. No. 33.

SCHWARTZ, D., FLAMANT, R., LELLOUCH, J. AND DENOIX, P. F.—(1961) *J. natn. Cancer Inst.*, **26**, 1085.

SEGI, M., FUKUSHIMA, S., FUJISAKU, S., KURIHARA, M., SAITO, S., ASANO, K. AND KAMOI, M.—(1957) *Gann*, **48**, Supplement.

" Smoking and Health "—(1964) Report of Advisory Committee to the Surgeon General of the Public Health Service, Washington, D.C.

STASZEWSKI, J.—(1960) *Br. J. Cancer*, **14**, 419.—(1961) *Polski Tygod. lek.*, **16**, 287.—(1964) *Br. J. Cancer*, **18**, 1.—(1966) *Br. J. Cancer*, **20**, 32.

" The Health Consequences of Smoking. A Public Health Service Review: 1967." U.S. Department of Health, Education and Welfare, Public Health Service Publication No. 1696, revised January, 1968, Washington D.C.

WYNDER, E. L., KMET, J., DUNGAL, N. AND SEGI, M.—(1963) *Cancer, N.Y.*, **16**, 1461.

WYNDER, E. L. AND SHIGEMATSU, T.—(1967) *Cancer, N.Y.*, **20**, 1520.